# Select and Cluster: A Method for Finding Functional Networks of Clustered Voxels in fMRI

**DOI:** 10.1155/2016/4705162

**Published:** 2016-09-05

**Authors:** Danilo DonGiovanni, Lucia Maria Vaina

**Affiliations:** ^1^ENEA, Unità Tecnica Fusione, Via Enrico Fermi, 45 00040 Frascati, Italy; ^2^Department of Biomedical Engineering, Boston University, 44 Cummington Mall, Boston, MA 02215, USA; ^3^Department of Neurology, Harvard Medical School, Massachusetts General Hospital, 175 Cambridge Street, Boston, MA 02114, USA

## Abstract

Extracting functional connectivity patterns among cortical regions in fMRI datasets is a challenge stimulating the development of effective data-driven or model based techniques. Here, we present a novel data-driven method for the extraction of significantly connected functional ROIs directly from the preprocessed fMRI data without relying on a priori knowledge of the expected activations. This method finds spatially compact groups of voxels which show a homogeneous pattern of significant connectivity with other regions in the brain. The method, called Select and Cluster (S&C), consists of two steps: first, a dimensionality reduction step based on a blind multiresolution pairwise correlation by which the subset of all cortical voxels with significant mutual correlation is selected and the second step in which the selected voxels are grouped into spatially compact and functionally homogeneous ROIs by means of a Support Vector Clustering (SVC) algorithm. The S&C method is described in detail. Its performance assessed on simulated and experimental fMRI data is compared to other methods commonly used in functional connectivity analyses, such as Independent Component Analysis (ICA) or clustering. S&C method simplifies the extraction of functional networks in fMRI by identifying automatically spatially compact groups of voxels (ROIs) involved in whole brain scale activation networks.

## 1. Introduction

Several data-driven techniques have been proposed for extracting functional connectivity patterns among cortical regions in fMRI datasets [1, 2]. These techniques can be grouped into three main categories: (i) methods based on pairwise measurements of connectivity between spatially segregated locations [3–5]; (ii) methods based on eigenimages decomposition of the image series into main components (PCA, ICA) [6–9]; (iii) clustering methods which group voxels on the base of a similarity distance and resulting in distinct functional clusters.

The pairwise measures have the advantage of being easily interpretable and benefit of a robust univariate framework for assessing significance. However, they are quite sensitive to noise and outliers and are not well suited for whole brain connectivity analysis because the global connectivity patterns are usually fragmented over a large number of pairwise relationships. Among pairwise measures, correlation analysis is one of the most widely exploited tools for studying interactions among brain areas [10, 11], since it is strictly related to the common definition of functional connectivity as quantifying temporal correlations between spatially segregated areas. It also provides a simple framework for the assessment of statistical significance [12] and similar to other data-driven methods it does not require a priori definition of a model of interaction between brain areas. Its major drawback, however, is that it is unpractical to use for a whole brain connectivity study given the high number of significant connections that are usually found. A solution to this problem is to limit connectivity analysis to a set of reference ROIs whose spatial position and extension are derived from brain activations and the fMRI literature [13]. However, in taking this approach networks not including the chosen seed reference ROI are not accounted for. To overcome this limitation while still performing a whole brain analysis, an alternative solution is to downsample the brain volume to get a smaller set of the time series from the mean signals of spatially contiguous voxels and apply correlation analysis on this set. Some of the proposed downsampling solutions are based on anatomical parcellation either exploiting information provided by a Brain Atlas or based on a clustering procedure in the anatomical space [14, 15]. Anatomical knowledge based methods make the assumption that voxels from the same anatomical area are also functionally related. To relax this strong assumption, it has been proposed to take functional information into account in the parcellation [16], but this approach requires a priori assumptions on the number of areas to be derived in the parcellation and also to perform a priori modeling of functional activations responses in the tasks presented in fMRI.

Exploratory methods of functional connectivity based on eigenimages decomposition (ICA or PCA) are a powerful tool for extracting the main sources of variance in the data and provide a global overview of functional relationships among brain areas (ROIs). However, a drawback of such methods is that they lack a clear framework for assessing statistical significance of the spatial maps for each component, even though several probabilistic models have been proposed for pattern-level noise-rejection criteria [17]. Furthermore, these methods are not suitable for classifying the set of components resulting from the analysis into components of interest and noise components either by paring the spatial component associated time-series with expected activation patterns or looking for consistent patterns over multiple subjects within the group level analysis [8, 18]. As a consequence, the spatial segmentation of the brain volume in functional ROIs obtained by thresholding the spatial maps of regions of interest depends on the choice of both the map and the threshold [7].

Finally, the clustering methods [19–25] also have problems to identify patterns of activation and specific ROIs since they force voxels to be grouped into separate functional clusters. A clear segmentation in spatially compact ROIs is often not possible since no spatial information is taken into account and spatial boundaries within a component are usually not smooth, unless an a priori functional model is imposed which provides voxels partitioning [16]. Moreover, important parameters such as the number of clusters to be defined are chosen by ad hoc or post hoc procedures.

In this paper we propose a new data-driven exploratory method, the Select and Cluster (S&C). The S&C method receives as input a single subject's fMRI dataset and provides as output the set of all ROIs involved in any pattern of significant functional connectivity present in the data with no assumptions on the number, position, shape, and size of the ROIs or on the expected functional profile generated by subjects' response to the task presented during fMRI scanning. We define these automatically extracted ROIs as* connectivity ROIs*: spatially compact groups of voxels showing a homogeneous pattern of significant connectivity to other regions in the brain [26]. Since the set of connectivity ROIs automatically extracted by S&C can be very large, we also propose a postprocessing step to visualize in a network the subset of extracted ROIs and connected to a seed ROI chosen from the set. These* connectivity networks* can then be compared to those obtained by other methods, such as ICA, PCA, or clustering methods.

The S&C method is composed of three main macrosteps: (a) starting with the entire cortex all voxels showing statistically significant correlation to any other voxels are extracted, while limiting the occurrence of spurious correlations through applying a multiresolution correlation analysis at two spatial scales; (b) a low-dimensional representation of significantly connected voxels time series is extracted; (c) voxels are grouped through a clustering procedure based on a spatiofunctional metric defined in a feature space given by spatial coordinates and functional weights of the low-dimensional representation calculated in (b). The output of spatiofunctional clustering step is therefore a set of ROIs involved in connectivity networks. The final postprocessing step is a procedure based on maximal cliques analysis [27] by which the set of ROIs produced in the output are grouped by the clustering step into networks of mutually significantly connected ROIs.

We will show that the S&C algorithm merges into a unique methodology, which has the most advantages of comparable approaches. In particular, it shares with linear pairwise measure (as the Pearson Correlation coefficient) the simple interpretability and robust statistical inference. It exploits the compact and noisy-less representation of main functional patterns in the data provided by eigenimages decomposition methods. Finally, it provides a spatial segmentation of the brain based on the SVC clustering which takes into account both the functional profile of the voxels and their spatial position without requiring any assumption on the ROIs number, size, or shape.

The document is organized as follows: Section 2 presents materials and the S&C method and Section 3 describes the validation of the method and its comparison to other methods available in literature. In particular in Section 3, (i) the S&C spatiofunctional clustering efficacy is compared to other clustering methods available in literature on a public hybrid dataset, (ii) the ability of S&C method to automatically extract ROIs from the data without losing signal of interest is validated on an experimental fMRI dataset on which also a set of reference ROIs has been independently extracted exploiting standard activation analysis methods, and (iii) the efficacy of S&C at extracting connectivity networks involving a chosen seed ROI is compared to ICA on a sample subject from the experimental dataset.

## 2. Material and Methods

In this section the Select and Cluster (S&C) algorithm is described in full detail.

### 2.1. Select Step: Multiscale Correlation Analysis (MSCA)

**Figure 1:**
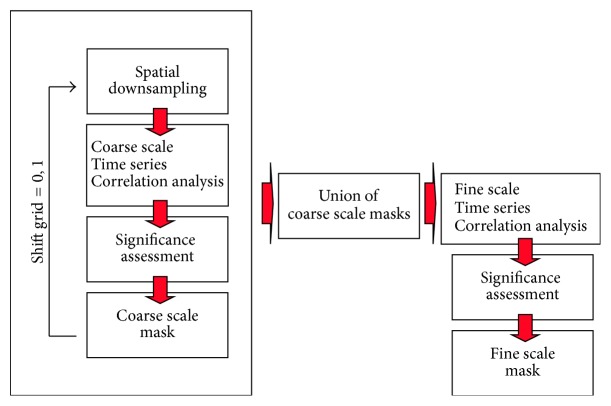
Schematic representation of the multiscale correlation analysis algorithm aimed at selecting those Grey Matter voxels showing significant connectivity to be used for further analysis.

The first step in the S&C method is the multiscale correlation analysis outlined in Figure 1. This step implements the* Select* part of the algorithm, aimed at extracting the subset of voxels significantly correlated to any other cortical voxel from the set of all voxels in the cerebral cortex (≈10^5^ voxels per brain volume). To deal with curse of dimensionality and the presence of spurious signals we implemented a 2-spatial scales correlation analysis: a coarse scale correlation analysis, performed on average signals of groups of neighbor voxels, is followed by a fine scale correlation analysis, performed at the scale of fMRI data acquisition voxels. For not biasing the analysis with the arbitrary voxel grouping method, the downsampling methodology we have adopted here does not rely on any a priori anatomical or functional information.

**Figure 2:**
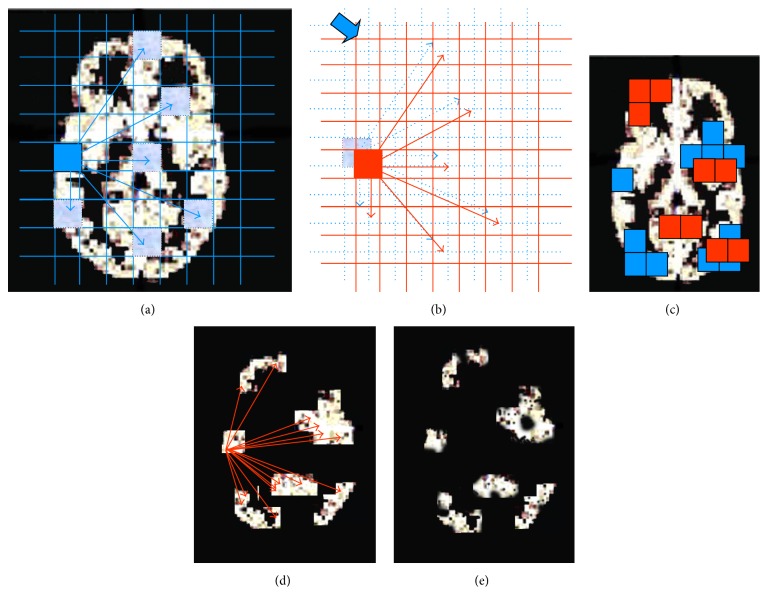
2D visualization of the 3D multiscale correlation analysis procedure implemented. (a) Coarse voxels time series are derived from fine scale cortical voxels and an all to all correlation analysis is performed; (b) the downsampling coarse grid is shifted and the correlation analysis is repeated on the corresponding new set of coarse voxels time series; (c) a Binary Mask is derived from the union of all coarse voxels (blue squares from first correlation analysis and red squares after the grid shift) involved in at least one significant connection in one of the two coarse level correlation analyses. (d) Fine scale correlation analysis performed on the set of voxels in the output from the coarse scale correlation analysis. (e) A fine scale binary mask derived from the union of all cortical voxels involved in at least one significant connection at the fine scale (in black are the voxels eliminated from the correlation).

A 2D visualization of the multiscale correlation analysis procedure is shown in Figure 2. The signal associated to coarse voxels in each scan is extracted to create* coarse scale* time series and a coarse scale correlation matrix is then calculated. Significant pairwise correlations are then identified in the correlation matrix according to the procedure described in Appendix A.1. A Coarse Binary Mask is then derived from the union of all coarse voxels involved in at least one significant connection. With reference to the algorithm summarized in Figure 1, to avoid signal loss due to the arbitrary initial positioning of coarse scale downsampling grid (Figure 2(a)), a second coarse correlation analysis iteration is performed after shifting in every dimension the coarse grid by 1 voxel at the acquisition scale (Figure 2(b)). The union of the two Coarse Binary Masks (Figure 2(c)) is then exploited to select the acquisition scale voxels which enter a second correlation analysis step (Figure 2(d)). Again correlations from this second analysis are tested for significance and selected to enter the clustering step of the S&C method.

Since in the coarse scale correlation analysis we consider all coarse voxel with at least 1 nonzero fMRI data acquisition scale voxel, to reduce the occurrence of outliers we require those voxels to have at least 4 significant correlations [3]. The 3-dimensional algorithm implemented is described in full detail in Appendix A.2. It is worth mentioning here that coarse correlation analysis is performed at the resolution element (*resel*) scale [29]; that is, coarse voxels are of the order of the Full Width at Half Maximum in mm of the Gaussian kernel used for spatial smoothing. This choice has been reported to provide the best tradeoff between downsampling and signal loss in anatomical parcellation applications [30].

### 2.2. Cluster STEP: Spatiofunctional Support Vector Clustering

The clustering part of S&C method is based on the Support Vector Clustering (SVC) algorithm which exploits a specifically designed spatiofunctional kernel adopted as the metric for the clustering process.

#### 2.2.1. Overview of Support Vector Clustering Algorithm

**Figure 3:**
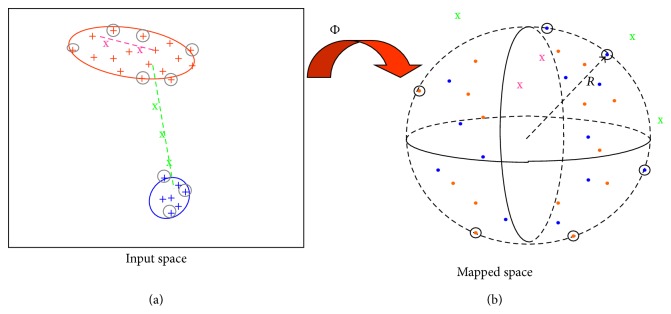
Graphical representation of Support Vector Clustering working principle: Support Vectors, marked with circles, define the boundaries of clusters in the input space (a) and lie on the surface of the minimal enclosing hyper sphere in the mapped space (b), while all other nonsupport vector input points will lie within the hyper sphere in the mapped space. Φ is the mapping function across input (a) and mapped space (b). A path connecting two input points belonging to the same cluster has been sampled in pink in the input space: points on this path will be mapped inside the hypersphere on the right. A path connecting two input vectors belonging to two different clusters has been sampled in green in the input space: points on this path will be mapped outside the hypersphere on the right.

The Support Vector Clustering (SVC) algorithm [31] is a particular application of the Support Vectors Machines (SVM) [32] classification algorithm. The key idea under SVC is that two input vectors belong to the same cluster only if no point on the straight path treaded between the two vectors falls out of the cluster boundaries (Figure 3, input space). To group input space vectors in clusters, the SVC algorithm maps these vectors into a high-dimensional space (Figure 3) where the support domain of the input points sample set can be described by the minimal enclosing hypersphere with radius* R*. The SVC key observation is that any point on the test path falling out of any cluster boundary in the input space falls out of the minimal enclosing hypersphere in the mapped space.

The SVC algorithm implements the clustering strategy described above in two steps:

(1) The first step is estimation of the domain support of the sample vectors [34] to find the minimal enclosing hypersphere* R*:

(1) R2x→ϕx→−a→2=Kx→,x→−2∑j=1NβjKx→j,x→+∑i=1N∑j=1NβiβjKx→i,x→j

with $\left\{\left.{\vec{x}}_{i}\right\}\subseteq \chi \right.$ composed of *N* points with *χ*⊆*ℜ* ^*d*^ representing the input space, *ϕ* a nonlinear mapping function from *χ* → *ℜ* ^*n*^ with associated kernel function* K* [33], such that $K\left({\vec{x}}_{i},{\vec{x}}_{j}\right)=\phi \left({\vec{x}}_{i}\right)\cdot \phi \left({\vec{x}}_{j}\right)$ and $\vec{a}$ is the center of the hypersphere. *β* parameters represent the Lagrange multipliers used in the optimization problem (Appendix A.3).

(2) The second is segmentation in disconnected regions by means of the boundaries of the domains. To assign different input vectors to different clusters a geometrical approach is exploited by sampling 20 points on the straight path connecting each couple of input points and verifying whether in such a path some points, ${\vec{y}}_{i}$, exist such that $R({\vec{y}}_{i})>R$.

This way the following adjacency matrix* A* between all couples of input points ${\vec{x}}_{i}$ and ${\vec{x}}_{j}$ is defined:

(2) $$\begin{array}{c} A_{ij}=\left\{\begin{array}{cc} 1⟺R\left(\vec{y}\right)\leq R & \forall \vec{y}\in \left\{path\left({\vec{x}}_{i}⟶{\vec{x}}_{j}\right)\right\}\\ 0 & otherwise. \end{array}\right. \end{array}$$

The clusters are defined as the connected components of the graph based on the matrix* A*.

#### 2.2.2. Application of SVC to ROIs Automatic Extraction from fMRI Data

In our application we will use the SVC algorithm to extract spatially compact groups of voxels which are also functionally homogeneous. Note that the ROIs and the functional cluster are defined on the basis of the voxels activation time series and S&C method. Other computational methods rely on the similarity of voxels activation pattern for clustering them into a single ROI. In the present work an attempt to relate these “activation based ROIs” to neurophysiological areas is made by labeling the reference ROIs used for method validation on the base of literature information to provide some link to anatomical identification of such ROIs.

We therefore need the SVC algorithm to cluster voxels and take into account both spatial and functional properties. The solution we adopted was the implementation of a spatiofunctional kernel to use in (3). In particular, since Gaussian kernels function are known to induce more compact support for the training data in the associated space [34] and since the product of two valid kernels is still a valid kernel [35], two Gaussian kernels were composed to give the following kernel expressing a dot product between two voxels *v*_*i*_ and *v*_*j*_:

(3) ksfvi,vj=e−∑k=1,…,3γksvixk−vjxk2e−∑p=1,…,PγpFviw~PCp−vjw~PCp2.

In (3) (see Appendix B for more detail),

(i) *x*_*k*=1,2,3_ ≡ (*x*_1_, *x*_2_, *x*_3_) = (*x*, *y*, *z*)_MNI_ are the MNI spatial coordinates of the voxels;
(ii) for a given voxel *v*_*i*_, ${\tilde{w}}_{PCp}\;$ represents the* t*-transformed weight for the* p*th component derived from a Singular Value Decomposition [36] of the time series of all voxels in output of the multiscale correlation analysis;
(iii) *P* is the number of principal components to use in the functional kernel and it was derived exploiting both the information theoretic estimate provided by the Minimal Description Length criterion [36] and the Akaike's Information Criterion (AIC) [37] across multiple subjects;
(iv) *γ* ^*S*_1,2,3_^ > 0 is a scaling parameter for spatial distances estimated as *γ*_*k*_^*s*^ = 1/*σ*_FWHM_*k*__^2^, *σ*_FWHM_*k*__ = FWHM_*k*_/2.35 for each spatial dimension *k*. The constant factor 2.35 is the proportionality factor between the sizes of FWHM (full width and half maximum) and sigma for a Gaussian. This choice allows us to express distances in units related to the actual spatial resolution determined by the amount of spatial smoothing;
(v) *γ*_*p*_^*F*^ is a scaling parameter for functional distances derived from the corresponding distributions of the principal components* t*-transformed weights as *γ*_*p*_^*F*^ = 1/*σ*_*l*_^2^, *l* = 1,…, *P*, where *σ*_*l*_ is the standard deviation estimated from the distribution of the *l*th component.

Once the spatiofunctional kernel for the SVC clustering algorithm is defined, when using SVC on a fMRI dataset, as the number of voxels to cluster increases, two problems can possibly occur: (i) the clustering process becomes computationally expensive (to calculate for each pair of voxels whether the path connecting them exits the candidate cluster support); (ii) the minimal enclosing hypersphere derived from (1) is less efficiently estimating the domain support for the set of voxels in input. To overcome these two problems and speed up the clustering procedure, a partitioning of the subject brains into coarse anatomical areas is performed* after* the multiscale correlation voxel selection procedure and* before* SVC clustering step. Therefore SVC algorithm groups voxels selected by multiscale correlation analysis and belonging to the same anatomical region. It is important to note that introducing this anatomical information after the blind multiscale correlation analysis step keeps the Select step of the S&C algorithm unbiased by a priori anatomical assumption for the number of voxels to retain but just can increase the number of ROIs obtained in output. The anatomical labeling method [38] from SPM2/5/8 was used, where a total of 45 Volumes of Interests are defined for each hemisphere.

### 2.3. Visualization of Connectivity Networks

The procedure summarized described in Section 2.1 gives as output a set of significantly correlated voxels which are grouped into ROIs with the procedure described in Section 2.2. Here we present a postprocessing procedure to extract networks of mutually connected ROIs:

(1) For each of the* n* ROIs obtained in output of the SVC, a mean time series is calculated and a pairwise correlation analysis is performed among all ROIs mean time series. Significance assessment is performed at a level of 0.05 corrected for multiple comparison using the Bonferroni correction.
(2) The correlation matrix is interpreted as an adjacency matrix *A*_*i*,*j*_ where *i*, *j* = 1,…, *n* has nonzero entries for all connected ROIs *i*, *j* and a search for* cliques* is performed in the undirected graph* G* associated with the adjacency matrix *A*_*i*,*j*_. Given a graph G, the* cliques* are defined as all possible subsets of vertices* V* such that, for every two vertices in* V*, there exists an edge connecting them. In our application each vertex is an ROI and each of the edges represents a significant connection the ROI forms with other ROIs.
(3) For each given ROI, its associated maximal clique (i.e., the largest set of mutually connected ROIs including that specific ROI) is interpreted as a connectivity network [39].
(4) All networks can be visualized by considering in turn each of the* n* ROIs obtained in output of the spatiofunctional SVC step.

The resulting networks are comparable to the output of other exploratory methods such as ICA.

### 2.4. Datasets

To validate our method we used two fMRI datasets. 

*Hybrid fMRI Dataset*. To prove the efficacy of the proposed clustering method based on the spatiofunctional metric induced by the kernel in Section 2.2.2, we tested its performance on a hybrid (synthesized) dataset exploited in [28] previously used for quantitative comparison of clustering methods applied to fMRI data. A hybrid dataset is obtained by taking superimposing artificial fMRI activation signal to a single slice extracted from real experimental fMRI data from a single subject. The data set consists of a time series of 140 time images of the single brain slice, with a matrix size of 64 times 64 pixels. The slice chosen was overlaid with 25 pixels of activation (a square of 5 × 5 pixels) with a contrast-to-noise ratio of 1.33, 1.66, and 2 where the noise was calculated within a cortical region. The hybrid datasets are ordered by increasing levels of contrast to noise ratio (CNR) and, respectively, labelled h4, h5, and h6 (for consistency with [28]). 

*In Vivo fMRI Data from Human Subjects*. We also used fMRI data from 8 right handed healthy subjects (ages 19–23) who performed three visual motion discrimination tasks. The data was acquired at the Athinoula Center for Biomedical Imaging, Massachusetts General Hospital, Boston. All subjects gave informed consent to participate in the research, according to the IRB requirements of Massachusetts General Hospital. The stimuli consisted of random dot kinematograms in which a proportion of the dots moved in the same direction while the rest provided masking motion noise. In the first task, Motion Coherence-radial (MCT) [40], the signal dots were moving radially. The subject's task was to determine the direction of the motion (expanding or contracting). In the second task, Motion Discontinuity (MDT) [40], subjects were required to determine whether the motion display was homogeneous or discontinuous. The discontinuous display was defined by an imaginary line in one of the following orientations: vertical, horizontal, and diagonal from upper right to lower left or from upper left to lower right. This line divided the motion display into two halves and it always intersected the display in the center. This imaginary line resulted from opposite directions of motion (up or down) of the signal dots in the two halves of the display. The third task, Motion Discontinuity defined Form from Motion (MDTPM) [40], was similar to the MDT task, except it portrayed a 2D form (cross or a bar) shown in at the center of the display. Here too, the forms were defined by an imaginary line defined by opposite directions of motion (up or down) of the signal dots within the shape area and outside (the background).

All tests were presented in a blocked design paradigm with temporally interleaved ON (30 s) and OFF (15 s) periods repeated in random order 6 times in a run, for a total of 285 sec (with an extra 15 sec OFF at the end). In all tests and all runs, the OFF periods consisted of a static random pattern display with the same statistical characteristics (aperture diameter, dots density, luminance, and size) as the motion displays.

*fMRI Data Acquisition and Preprocessing*. The fMRI data were acquired with a 3.0-T Magneton VISION (Siemens, Germany) whole-body MRI system equipped with a head volume coil. For the fMRI functional volume acquisition, an echo planar imaging (EPI) sequence sensitive to blood oxygen level dependent (BOLD) effects was used with following imaging parameters: repetition time TR = 2.5 s, echo time TE = 30 ms, flip angle of 90 deg, and field of view of 200 mm. The brain volume consisted of 22 slices of axial orientation, image size was 64 × 64 pixels, and slices thickness was 5 mm with a gap of 1 mm. Therefore, slices were covering the whole brain; voxel size of EPI images was 3.13 × 3.13 × 6.00 mm. For anatomical localization, 3D gradient echo T1-weighted sequence was used, with anatomical image size of 256 × 256 × 128 pixels, slices thickness of 1.33 mm, and voxel size of 1.00 × 1.00 × 1.33 mm.

In all subjects the retinotopic areas were defined by using the classical experimental paradigm for retinotopic mapping (rotating wedges and expanding rings). The data were preprocessed with the SPM2 package (http://www.fil.ion.ucl.ac.uk/spm/spm2.html).

After data preprocessing, a set of 16 subject-specific ROIs were defined in each hemisphere, on the base of single subject cortical activations that resulted from the three motion tasks (MTLOC, MDT, and MDTPM) and from the retinotopic mapping. The ROIs definition was furthermore checked against the existing fMRI literature [41]. These subject specific ROIs obtained with standard model based activation analysis were used as reference ROIs to validate the performance of proposed S&C method to automatically extract subject specific ROIs.

## 3. Results

### 3.1. Comparison of Spatiofunctional SVC with Other Clustering Algorithms

In this section, we first apply the Support Vector Clustering (SVC) algorithm and the spatiofunctional kernel (see (3)) on the hybrid dataset (described in Section 2.4). For each dataset, voxels outside the brain were masked out resulting in 1212 voxels to cluster. For datasets h5 and h6 the first 5 PC components were used in the functional kernel (second factor in (3)) while up to 7 components were considered for dataset h4, given the low CNR. Since no spatial smoothing preprocessing had been performed on this dataset, to calculate the *γ* ^*S*^ parameter according to (3), the FWHM (Full Width at Half Maximum) Gaussian kernel used for smoothing the data was estimated directly from data through the method proposed in [42].

Second, the SVC clustering performance is compared to the performance of the following methods: Hierarchical Clustering in the single linkage, complete linkage, and ward implementation [43];* K*-means algorithm; Fuzzy clustering [20, 22], Neural Gas [25], Self-Organizing Maps (SOM) [44]; Crisp clustering, based on maximum distance.

The evaluation of the performance of these clustering methods was done by using the weighted Jaccard coefficient (*w*_*JC*_), defined as *w*_*JC*_ = (*a* + *P*(*a*)^−1^)/(*a* + *P*(*a*)^−1^ + *b* + *P*(*b*)^−1^ + *c* + *P*(*c*)^−1^), where *a* is the number of True Positives, *b* the number of False Negatives, and *c* the number of False Positives voxels and *P*(*a*)^−1^, *P*(*b*)^−1^, and *P*(*c*)^−1^ are the inverse of corresponding probabilities [28]. Using weights based on inverse probabilities allows for rare True Positive class to be emphasized in the clustering quality assessment. The *w*_*JC*_ coefficient was calculated on the “activation” cluster, defined as the cluster for which the maximum absolute number of TP voxels was found by the algorithm.

In Table 1 we report performances of all clustering algorithms considered here on the three datasets with increasing CNR. Since SVC algorithm finds 10 clusters in each dataset h4, h5, and h6, respectively, we compare its performance to the performance of other methods (Table 1) for the number of clusters parameter set to 10. Since the number of clusters found by SVC nonlinearly depends on the values chosen for the spatiofunctional kernel *γ* parameters we checked the stability of SVC algorithm performance at their variation. Results shown in Table 1 using the estimators described in Section 2.2.2 are stable in a range $\tilde{\gamma }\pm 0.2$ around the proposed estimate value.

### 3.2. S&C Method Performance on Real fMRI Data

We apply the whole S&C method to a fMRI dataset acquired in our laboratory described in Section 2.4 to investigate the ability of S&C method to select voxels of interest and group them in ROIs. As benchmark method for ROIs definition we adopted a standard activation analysis based on* t*-test comparison of the ON and OFF conditions of the experimental paradigm (Section 2.4).

#### 3.2.1. Noise Reduction Properties of Multiscale Correlation Analysis Method (MSCA)

We will assess the noise reduction property of multiscale correlation procedure by showing that a more efficient extraction of the components of interest can be performed on the resulting reduced set of voxels. In particular, we will use Minimal Description Length (MDL) method to estimate the number of independent components conveying most of information included in the voxels time series. Only a part of these components will be labelled “component of interest” on the base of their similarity to the stimulus pattern. If multiscale correlation analysis is able to reduce the number of independent components (as estimated by MDL) while retaining those similar to the stimulus pattern, then we will have a demonstration of the fact that MCA rejects just noisy components.

Table 2 shows, for all subject and tasks, the number of components estimated with the Minimal Description Length (MDL) [45] on the reduced dataset resulting from the application of the multiscale correlation analysis. The number of components estimated on all cortical voxels is reported in brackets.

In Table 3 we present the stimulus related components extraction performance for the reduced set and the initial set of cortical voxels. Specifically, the mean correlation coefficient absolute value and the mean percentage of variance accounted by the stimulus related component are reported for the three sets of voxels across subjects and tasks. Tables 2 and 3 show that (i) the multiscale correlation analysis (MSCA) procedure is able to extract a subset of voxels whose activation patterns can be described using a small number of components putting in evidence the spatiotemporal covariance structure dimensionality reduction properties of the procedure and (ii) stimulus related components are better extracted in the reduced dataset with respect to all cortical voxels, suggesting that dimensionality reduction occurs at the expense of noise components.

In Table 4 we report the number of components used in the functional kernel for each subject across tasks. It is important to note that we must provide the functional kernel with an informative representation of the functional space and not to estimate the exact number of sources to obtain a correct decomposition, as suggested by other methods (as for ICA). As a tradeoff between the necessity of having a small number of clustering features and that of not losing possibly interesting signal, we decided to use, for each subject *i* in a given task, a number of components given by

(4) Psi=max⁡PsiMDL;meanmediani∈1,…,NPsiMDL,mediani∈1,…,NPsiAIC

that was extracted on the reduced dataset of voxels selected from the multiscale correlation analysis procedure. Moving from the assumption that the number of task-relevant sources of variance should be consistent across subjects, (4) then provides a better representation for those subjects having a too conservative MDL estimate of the number of components.

#### 3.2.2. Agreement between Reference Model Based ROIs and SVC Induced ROIs

To assess S&C method clustering performance we adopt the* external criteria* approach [46, 47], meaning that the performance is calculated with respect to some external reference clustering scheme. Specifically, we compare for each subject across tasks the grouping of voxels obtained by the spatiofunctional SVC method to the grouping of voxels in reference ROIs resulting from standard* t*-test on activation analysis. The Fowlkes-Mallows index as performance quality index was used for comparison. Let *C*_*m*_^*l*^ = {*C*_1_,…, *C*_*m*_} be the clustering partition of* l* voxels in* m* clusters, obtained after running the SVC clustering algorithm on the data set* X* of the* l* voxels in the predefined ROIs and showing significant connectivity. We then define *P*_*s*_^*l*^ = {*P*_1_,…, *P*_*s*_}, the reference partition of these* l* voxels into* S* predefined ROIs. We assigned a label to each pair of voxels (*v*_*i*_, *v*_*j*_)_*i*,*j*={1,…,*l*}_, derived from the data set* X*, according to the labelling criteria presented in Table 5.

**Figure 4:**
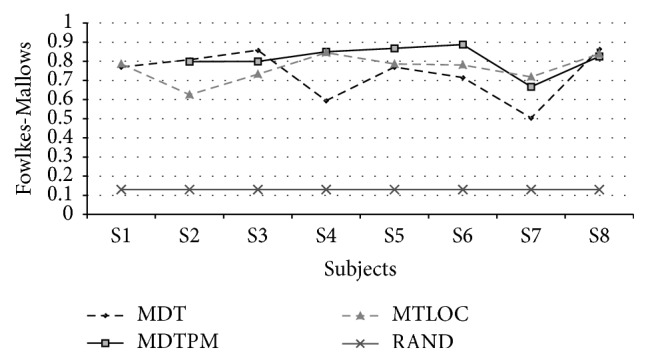
FM index relative to the compatibility test between the activation ROIs partition and the partition induced by the spatiofunctional SVC. Voxels used in the computation are those ROIs voxels resulting in significant connectivity as assessed by the multiscale correlation analysis (MSCA).

The Fowlkes-Mallows index (FM) is defined as $FM=\sqrt{\left(\left(a\left(a+b\right)\right)\left(a\left(a+c\right)\right)\right)}$ which can be interpreted as the geometric mean of (*a*(*a* + *b*)) that is the probability that two points belong to a same cluster in* C* if they belong to a same cluster in* P* and (*a*(*a* + *c*)), the probability that two points belong to a same cluster in* P* if they belong to a same cluster in* C*. This index ranges between 0 and 1, where 1 stands for perfect agreement between the clustering induced partition and the reference partition of the considered set of voxels. In Figure 4 the FM performance curves are reported for each motion discrimination task. To help interpretation of the measure of agreement between reference ROIs and correspondent SVC induced clusters, we also report the FM index value for a random labeling simulation. In particular, the mean FM value (0.13) was calculated over 10^4^ random labeling simulations of 200 voxels in* C* classes, where* C* was randomly chosen in each iteration from the set *C* = {1,…, 6*S*}.* S* indicates the true number of clusters in the reference partition. ROIs segmented through the retinotopic analysis were not considered in the performance assessment.

#### 3.2.3. Connectivity Networks of ROIs Found by S&C and Comparison to ICA

**Figure 5:**
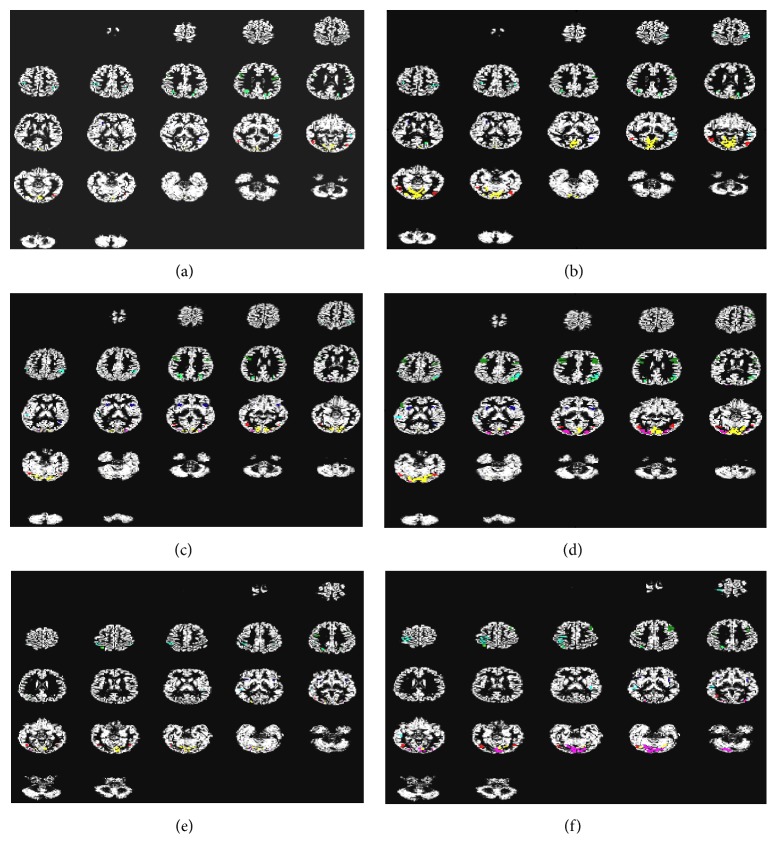
Predefined ROIs voxels showing connectivity are presented for three sample subjects in task MDT, MDTPM, and MDT, respectively (a, c, and e). Clusters induced by the spatiofunctional SVC clustering with maximal intersection with predefined ROIs are presented for three sample subjects MDT, MDTPM, and MDT, respectively (b, d, and f). Arbitrary colors are used to distinguish identified ROIs.

Figure 5 illustrates in three sample subjects a visual comparison between clusters induced by S&C method with maximal intersection with the predefined ROIs to the predefined ROIs that show significant connectivity in each of the motion tasks.

**Figure 6:**
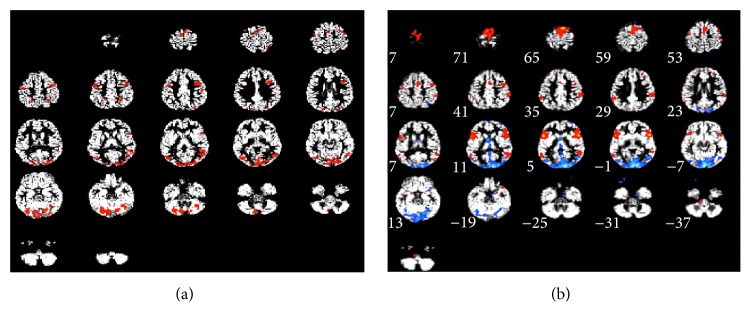
Comparison of ROIs clusters found by S&C method (a) and ICA method (b) for one sample subject in task MTLOC. (a) To generate the presented connectivity network map from our method's output we postprocessed the set of clusters in output by the SVC performing a pairwise correlation analysis on their associated mean time series. Then we extracted from the correlation matrix the largest set of all mutually significantly correlated ROIs including area MT in the considered subject. The resulting set of mutually connected ROIs was then visualized on the subject structural volume. To help visualization and comparison with ICA maps, all ROIs in the network are reported in same colour. (b) The first two stimulus related independent components, with Pearson Correlation coefficient with HRF of *R* = .42 and *R* = .31, are reported in blue and red colour, respectively. The independent components were* z*-transformed and a threshold of *z* ≥ 2 was applied.

Figure 6(a) shows for a sample subject the application of network visualization procedure of Section 2.3. In particular, we extracted the maximal clique network involving area MT in a sample subject for the MTLOC fMRI task. Since the MTLOC stimulus is designed to elicit activation in area MT [40] the maximal clique including area MT in Figure 6(a) can be interpreted as a network of coactivated ROIs responding to this stimulus. This allows us to compare the performance of our method at extracting networks of ROIs responding to the stimulus to that of other methods based on data decomposition, such as Independent Component Analysis [7, 8]. The GIFT toolbox (http://icatb.sourceforge.net/) was used to perform an ICA analysis on subject AB in the MTLOC task. The following parameters for decomposition were adopted: (i) number of components in the model was estimated as 27, exploiting the MDL principle (Section 2.2.2); (ii) The Informax algorithm [48] was used for decomposition. The independent components were then ranked according to the temporal correlation of their associated temporal profile with the stimulus HRF time profile. The first two components in this rank had Pearson Correlation coefficient with stimulus time profile of *R* = .42 and *R* = .31, respectively. These two components were* z*-transformed, thresholded at *z* ≥ 2 [7], and reported in (Figure 6(b)) in blue and red color, respectively.

## 4. Discussion

Section 3 showed S&C method ability in extracting directly from data: (i) a set of functionally homogeneous and spatially compact ROIs and (ii) visualizing the functional connectivity networks in which they are involved. These ROIs can be interpreted as connectivity ROIs [49].

The multiscale correlation analysis step (Section 2.1) reduces both the dimensionality of the problem and the incidence of local noise variance on the extraction of connectivity patterns as shown in Table 2. Moreover, dimensionality reduction only occurs at the expense of the noise components as shown in Table 3, where stimulus related components are more efficiently extracted from the reduced set of voxels than from the initial set of all cortical voxels. The MSCA makes a whole brain analysis feasible while avoiding bias possibly introduced by the definition of a seed ROIs [11, 13]. Moreover the approach makes no assumptions either for anatomically based downsampling [14, 15] or to preselect voxels for clustering analysis [50, 51] or about expected stimulus induced response. Moreover, the multiscale correlation procedure provides a simple and robust framework for statistical significance assessment. However, one limitation of exploiting correlation analysis as a measure for functional connectivity is the need to correct for datasets global noise derived from physiological noise and motion correction preprocessing steps.

Support Vector Machines have been proven to be effective in fMRI data analysis applications, for mental states classification [52, 53] or brain activity detection [54]. In the proposed S&C method we adopt a clustering purpose variant of Support Vector Machine theory, the Support Vector Clustering (SVC) algorithm, and exploit it to group preselected voxels into spatially compact and functionally homogeneous ROIs. Therefore, the strategy is different from that of methods [55] exploiting SVM to separate active voxels from nonactive ones. Moreover, we do not need a complex feature selection strategy for SVM [56] since selection of the subset of voxels of interests is accomplished by the multiscale correlation analysis, and the Minimal Description Length (MDL) principal component selection step is just needed to ensure minimal but congruent representation of patterns in the data, not to eliminate spurious signals. Moreover, we include the spatiofunctional information used in the clustering decision, directly in the proposed spatiofunctional kernel. The SVC clustering algorithm is able to select spatially compact and functionally homogeneous ROIs without requiring a priori assumptions on ROIs number, shape, and size as in other clustering methods. The global optimization problem solved in the SVC computation also avoids the problem of local minima solutions. According to the rank based on a weighted Jaccard coefficient (Table 1), the proposed spatiofunctional SVC clustering method performed better than other clustering methods on a public hybrid dataset (Section 3.1). With respect to other approaches [57, 58] the SVC clustering strategy greatly simplifies the exploitation of local spatial connectivity and activity/background contrast in the cluster segmentation process. In fact, SVC algorithm focuses on the detection of cluster border discontinuities and the implicit use of spatial and functional information provided by the kernel. Furthermore, the nonlinear mixing of all information conveyed by spatiofunctional features operated by the kernel allows the SVC to deal with inefficient PC decomposition, provided that the information of interest is still available in the exploited functional components. This tolerance of redundancy in SVC makes this algorithm less sensitive to misspecification of the number of components, which in the ICA method, for example, can cause the split of stimulus related variance over several components (Figure 6(b)). On the other hand, if insufficient information is conveyed by the principal components selected as clustering functional features, no cluster segmentation occurs, and the SVC produces no activation/background segmentation at all. This behaviour is also observed when no discontinuity in activation profile is registered at the spatial scale specified by the spatial kernel *γ*_*S*_ parameter, as it was shown in Figure 5(b) for reference visual areas in the occipital cortex which are grouped in a single cluster.

When applied to the fMRI data from the three visual motion tests, on the voxels selected by multiscale correlation analysis, the spatiofunctional SVC grouped the subset of voxels belonging to reference ROIs consistently, as quantified by Fowlkes-Mallows clustering quality index (Figure 4) and visually shown in Figure 5. Note that SVC reproduces reference ROIs in a completely data-driven way, that is, making use of no a priori information about the expected response temporal profile.

It should be noted that SVC computational cost is very high (several hours per subject) and given the clustering strategy based on edge segmentation, it can suffer from poorly defined support domain (Section 2.2). For this reason a coarse anatomical labeling procedure was implemented as a preprocessing step for SVC clustering on motion battery dataset. Notice that coarse anatomical labeling has no effect on the select step (it comes after the multiscale correlation analysis) and just provides SVC with more compact spatial domains in which to operate the voxels spatiofunctional grouping while not limiting its ability to extract ROIs. In fact, as shown in Figure 4, SVC grouping of the voxels is far better than a random grouping within the same anatomical area. Nevertheless, it would be interesting in future work to explore the robustness of spatiotemporal Support Vector Clustering step with respect to the coarseness of chosen anatomical parcellation method (Section 2.3).

The output of S&C method is a set of data-driven connectivity ROIs, which are organized in networks not necessarily mutually exclusive. The visualization procedure proposed in Section 2.4 focuses on the search of the maximal set of mutually correlated ROIs including a seed area, as shown in Figure 6 considering MT are in the task MTLOC, explicitly designed to elicit activation in this area. While this approach has similarities to previous studies that extract network of voxels significantly correlated with a specified seed ROI [11, 13], there are important differences: (i) the correlation analysis is defined on ROIs automatically extracted from fMRI data with the S&C procedure; (ii) the seed ROI is chosen a posteriori from the set produced by S&C method and can be changed to visualize all networks extracted from fMRI dataset with the data-driven procedure S&C.

The network obtained by our method (Figure 6) is in good agreement with that obtained by an ICA analysis on same subject when considering stimulus related components, with the advantage of not requiring a component of interest selection. Even though further work is needed to improve the extraction and visualization of mutually exclusive networks of ROIs from the methods output, the example provided in Figure 6 suggests that the proposed method can be successfully used for the detection of networks of coactivated ROIs, automatically segmented exploiting information available in the data.

Finally, the proposed use of kernel methods and Support Vector Clustering makes the clustering step very flexible and useful in the exploration of additional features [59] for voxels properties characterization, which may improve the sensitivity of the method. In future work we will address the problem of characterizing and validating across subjects [60] the ROIs networks identified in the analysis, with the interesting perspective of relaxing [16] ROIs spatial superposability assumptions across subjects.

## 5. Conclusion

In this paper we proposed a novel data-driven method, the S&C method, which extracts, without relying on any a priori knowledge, a set of ROIs mutually involved in connectivity patterns present in fMRI data. The S&C method efficacy was validated on a real fMRI dataset of subjects performing visual tasks. A set of reference ROIs has been defined exploiting commonly used model based activation analysis. The results proved that the multiscale correlation analysis procedure, the selection step of S&C method, is effective at reducing the dimensionality of the problem and extracts a subset of cortical voxels without signal loss. The spatiofunctional SVC algorithm, the clustering step of S&C method, has been validated on a public hybrid dataset (from [28]) on which the SVC algorithm demonstrates better performance compared to other commonly used clustering algorithm which was reported.

When applied to the real fMRI dataset the spatiofunctional SVC could reproduce consistently the grouping of voxels belonging to the reference ROIs, without relying on a priori information. The S&C method performance on extracting networks (computing connectivity) involving arbitrarily chosen ROI on real fMRI dataset was compared to ICA method. We obtained good agreement between the two methods, but the S&C method required less assumptions and resulted on better definition of the ROIs. The Select and Cluster method proposed is a promising, robust tool for localizing spatially compact and functionally homogeneous ROIs involved in significant functional connectivity and visualizing the networks of connectivity to which they belong.

## Figures and Tables

**Figure 7:**
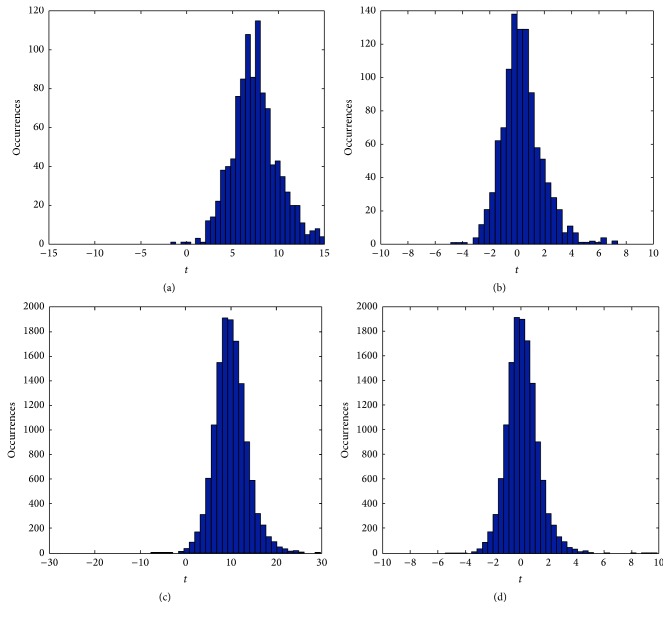
*t*-transformed correlation coefficient distributions at coarse and fine scale for a sample seed voxel in a sample subject before and after applying the correction. Coarse level* t*-transformed cross-correlation distribution (a) before applying normality correction and (b) after applying it. Fine scale* t*-transformed cross-correlation coefficient distribution (c) before applying normality correction and (d) after applying it.

**Figure 8:**
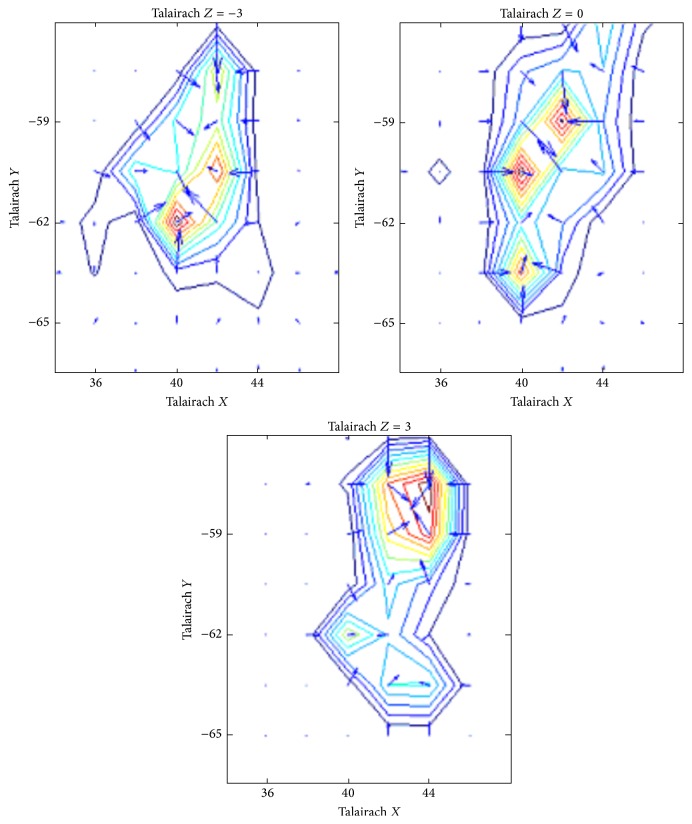
In the figure in Talairach* X*,* Y*, and* Z* coordinates, the spatial gradient of the number of pairwise connections to all other cortical voxels is reported, projected by voxels within a cubic volume centered in area MT right hemisphere for a sample subject in task MTLOC. The number of connections formed increases as reaching the center of MT area.

**Figure 9:**
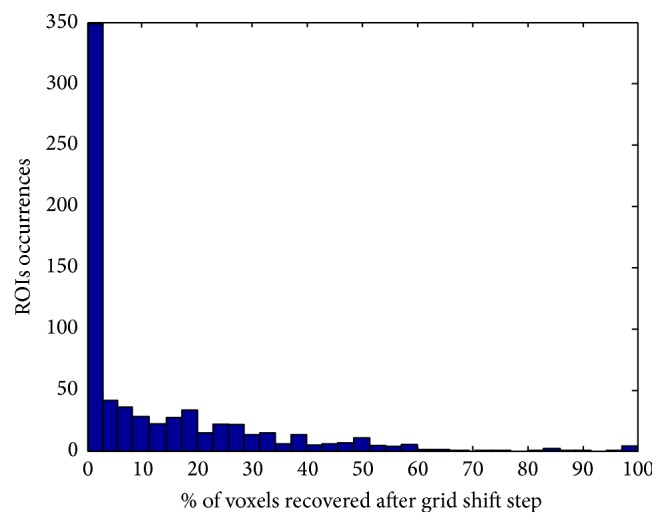
Percentage of voxels recovered after applying the coarse grid shift procedure to the total number of voxels in the ROI. Data from all subjects in all tasks were used to build the histogram. For some ROIs almost all voxels (98%) are recovered after the shift.

**Table 1:** Performance on a reference hybrid dataset with increasing CNR (h4 = 1.33; h5 = 1.66; h6 = 2), of the clustering methods used for fMRI data. The value of the weighted Jaccard coefficient is reported for the cluster with maximal number of True Positive voxels found by each method. Numbers in table (the highest the better) measure the number of voxels initially belonging to true cluster (artificially defined) which are put in the same cluster by each compared method. Since Support Vector Clustering resulted in a number of clusters ≤ 10 in each dataset, its performance is compared to other methods performance with the number of clusters initialized to 10. For nonhierarchical methods, clustering solutions depend on initialization conditions, so the mean performance across 50 different initialization conditions is reported. Note that the higher the *w*_*JC*_ performance score, the better the quality of the activation cluster. SVC method shows a stable performance across increasing levels of noise and performs better than other methods, partially adapted from Dimitriadou et al. 2004 [28].

		Hybrid dataset h4	Hybrid dataset h5	Hybrid dataset h6
Hierarchical	SVC	.96	.96	.96
	Hierarchical (ward)	.74	.89	.96
	Hierarchical (complete link)	.07	.06	.45
	Hierarchical (single link)	.01	.01	.01

Nonhierarchical	Fuzzy *C*-means	.67	.66	.67
	*K*-means	.63	.96	.96
	Neural gas	.30	.96	.96
	SOM	.68	.66	.67
	MaxiMin	.04	.04	.04

**Table 2:** Number of sources estimated with the Minimal Description Length criterion on the reduced dataset of voxels selected from the multiscale correlation analysis procedure. In brackets we report the number of sources estimated on the dataset of all cortical voxels. Subjects (S1 to S8) are in columns and tasks are in rows.

	S1	S2	S3	S4	S5	S6	S7	S8
MDT	2 (26)	3 (26)	2 (26)	6 (26)	5 (26)	7 (26)	2 (26)	3 (26)
MDTPM		3 (28)	3 (28)	4 (28)	5 (28)	6 (28)	5 (28)	4 (28)
MTLOC	4 (27)	4 (27)	9 (27)	4 (27)	5 (27)	7 (27)	4 (27)	4 (27)

**Table 3:** Comparison of SVD results for the set of ROIs voxels, the reduced set from multiscale analysis, and the whole cortex voxel dataset. For the three tasks and all subjects the maximally stimulus-correlated component was extracted and the following descriptive values were calculated: (i) mean correlation coefficient absolute value; (ii) mean percentage of variance accounted by the component. The descriptive values mentioned were calculated on two sets of voxels, respectively, before (all cortical voxels) and after multiscale correlation analysis method (reduced set of voxels).

		Mean rank	Mean corr to HRF	Mean var explained%
MDT	Reduced set of voxels	1.63	0.78	17.18
	All cortical voxels	2.13	0.72	14.75

MDTPM	Reduced set of voxels	1.86	0.71	18.21
	All cortical voxels	1.86	0.68	16.99

MTLOC	Reduced set of voxels	3.00	0.47	11.61
	All cortical voxels	3.50	0.46	9.06

HRF: hemodynamic response function.

**Table 4:** Number of sources estimated across subjects and tasks using (4) on the reduced dataset of voxels selected from the multiscale correlation analysis procedure. Subjects (S1 to S8) are in columns and tasks are in rows.

	S1	S2	S3	S4	S5	S6	S7	S8
MDT	5	5	5	6	5	7	5	5
MDTPM		6	6	6	6	6	6	6
MTLOC	6	6	9	6	6	7	6	6

**Table 5:** Pairs of voxels are classified into 4 classes depending on the relative grouping of constituting voxels in the reference partition and clustering scheme induced by the algorithm.

Partition of {(*v*_*i*_, *v*_*j*_)}	Same cluster in *P*	Different clusters in *P*
Same cluster in *C*	*a*	*c*
Different clusters in *C*	*b*	*d*

## Appendix

### A. **Additional Details on the MSCA Procedure**

In this appendix we first provide detailed, step by step, information for the significance assessment of the multiscale correlation algorithm (MSCA); second, we illustrate the spatial downsampling algorithm for the multiscale correlation analysis (MSCA) and third, we discuss several scenarios for downsampling validation.

#### A.1. Significance Assessment for Multiscale Correlation Algorithm (MSCA)

To assess significance at both scales, correlation coefficients *r*_*i*,*j*_ between each two voxels *i*, *j* = 1,…, *N*_*vox*_ were* t*-transformed through $t_{i,j}=r_{i,j}\sqrt{v}/\sqrt{1-{r_{i,j}}^{2}}$, where *ν* is the number of degrees of freedom. The presence of autocorrelation in fMRI time series [61, 62] will generally result into* t*-transformed cross-correlation distributions (of each voxel to the rest of the cortex at both scales considered) with nonzero mean and unit variance (Figure 7).

To correct for this effect, we applied the empirical methodology exploited in [11, 13]. Assuming that the* t*-distribution consists of a Gaussian distributed component from bulk system and physiologic correlations plus a tail from higher order correlations, a three-parameter least-square fit of the* t*-distribution from each voxel is made to a Gaussian function. The free parameters are the mean, standard deviation, and area. The fit is restricted to the full-width at half-maximum (FWHM) of the* t*-distribution of all connections projecting from each voxel *v*_*i*_0__ to all other voxels (we then have *N*_*vox*_ of such distributions), *t*_*i*_0_*j*_, and it is required to have a *χ* ^2^ probability greater than 0.05. Once the *μ*_*i*_0_*j*_ and *σ*_*i*_0_*j*_ are estimated for each seed voxel the distribution is corrected according to

(A.1) $$\begin{array}{c} {tcorr}_{i_{0}j}=\frac{t_{i_{0}j}-\mu_{i_{0}j}}{\sigma_{i_{0}j}}. \end{array}$$

After applying the correction, the resulting distribution is approximately standard normal (Figures 7(b) and 7(d)). At fine scale, the correction is calculated just for voxels surviving the mask, but to build the distribution correlations of the given seed voxel to all initial voxels in the cortex are considered.

Since spatial smoothing performed in the preprocessing alters local cross-correlation structure, correlations between neighbor voxels at acquisition scale are not considered. At coarse scale we correct the effect of spatial smoothing by requiring the distribution of correlations between neighbor coarse voxels after smoothing to match mean and variance of the correspondent distribution derived from same scans before spatial smoothing preprocessing step.

The significance level is controlled at *α* = 0.025 for coarse scale correlation analysis, accounting for the fact that two iterations are performed while it is controlled at *α* = 0.05 for acquisition scale correlation analysis. To address the problem of multiple comparisons we used the False Discovery Rate (FDR) approach described in [63].

In particular given* n* hypotheses to test and set the significance level to*α*, the following procedure was implemented:

(1) All* n* tests were ordered by increasing *P* values *P*_1_ < *P*_2_ < ⋯<*P*_*n*_.
(2) The number of null hypotheses to be rejected was given by
  (A.2) $$\begin{array}{c} N_{significant}=\max\left\{\;i:P_{i}\leq \frac{i}{n}\frac{\alpha }{\sum \left(1/j\right)}\;\right\}, \end{array}$$

where* n* was equal to *N*_*c*_*s*^2^__ − *N*_*c*_*s*__ and *N*_*F*_*s*^2^__ − *N*_*F*_*s*__ for coarse and fine scale, respectively.

#### A.2. Spatial Downsampling Algorithm for the Multiscale Correlation Analysis (MSCA)

The fMRI voxels acquisition grid provides a coordinate system in the cerebral cortex. To obtain coarser scale fMRI signal we consider a coarser scale grid where nodes centers are placed in the three dimensions as shown in the formals below:

(A.3) Vcsi,j,k=Cs·i−int−∞NxCs+Shift,Cs·j−int−∞NyCs+Shift,Cs·k−int−∞NzCs+Shift,i=1,…,int−∞NxCs;  j=1,…,int−∞NyCs;  k=1,…,int−∞NzCs,  Cs=int−∞FWHMsizeVoxel+1,  Shift=0,1,

where *C*_*s*_ is the parameter controlling the coarseness level of the downsampling procedure while the operator int_−*∞*_(·) rounds its operand to the first integer towards minus infinity. The scale parameter has been chosen equal to *C*_*s*_ = int_−*∞*_(FWHM/size(Voxel)) + 1, where FWHM is the Full Width at Half Maximum in mm of the Gaussian kernel used for the spatial smoothing preprocessing of each functional image. This choice for *C*_*s*_ allows us to obtain coarse voxels of the order of the FWHM of the spatial smoothing kernel. After placing the coarse grid nodes at points resulting from applying (A.3) on the data acquisition grid, in each functional scan, we take the average over the spatial volumes (referred to as* coarse voxels*) centered at nodes defined above and of size equal to *C*_*s*_. Note that only cortical voxels contribute to the average signal associated with each coarse voxel.

As an example, considering an acquisition voxel size of 3*∗*3*∗*6 mm^3^ and functional volumes composed of 22 slices of 64 by 64 pixels each, each volume would consist of (*N*_*x*_ *∗N*_*y*_ *∗N*_*z*_) = (64*∗*64*∗*22) = 90112 voxels. Then considering a spatial smoothing filter of FWHM of 6*∗*6*∗*12 mm, that is, 2 times the acquisition voxel size, we get *C*_*s*_ = 3 and a coarse voxel size of 3 times the acquisition voxel size in each dimension.

Figure 8 illustrates the rationale for a coarse scale correlation analysis to work as a preliminary dimensionality reduction step. The S&C method assumption is that for an ROI the connectivity signal is present over several spatially contiguous voxels and it increases when approaching the geometrical center of that ROI. Figure 8 shows the spatial gradient of the number of significant correlations per voxel within a cubic volume centered on area MT for a sample subject.

We note that redundancy is necessary for the blind sampling procedure to work. As seen in Figure 9, in the reference ROI a large number of voxels discarded by the* BLIND-DOWNSAMPLING* in the first iteration are recovered and selected for the following step of clustering analysis just after the coarse grid shift procedure.

#### A.3. Downsampling Validation Scenarios

*MODELBASED-DOWNSAMPLING* method exploits functional information-driven downsampling: a mean time series is derived for each subject specific ROI by averaging signals across all voxels belonging to that ROI. Subsequently a cross-correlation analysis was conducted among the time series of all ROIs. All ROIs involved in any significant correlation at this coarse level are used to build a binary mask, so that all voxels belonging to the significantly (significance level controlled at *α* = 0.05) connected ROIs enter a second correlation analysis on the voxels time series. The correction shown in (A.1) in this appendix was implemented by estimating parameters on the cross-correlation distribution of each ROI mean signal to the rest of cortical voxels.

*BLIND-DOWNSAMPLING* a coarse scale correlation analysis is performed on all cortical voxels as described in Section 2.1 in the paper. A mask is applied to retain only correlations among the coarse scale voxels intersecting the predefined ROIs. After the correction in (A.1), significance of correlations is assessed as explained in Section 1 of this appendix, by using the False Discovery Rate at a level *α* = 0.025 (accounting for the shift) and corrected for multiple comparisons with a* number of tests = n_coarse_roi* ^*2*^ *-ncoarseroi*. The* ncoarseroi n_coarse_roi* is the number of coarse scale voxels with nonnull intersection with reference ROIs. A binary mask is then derived from the union of all coarse voxels involved in any significant correlation and a second correlation analysis is performed on all voxels surviving the mask.

*NO-DOWNSAMPLING* is described as follows: To have a reference on the signal loss occurring when performing a multiscale correlation analysis with the methods above, we also performed a correlation analysis directly on the whole set of voxels in the reference ROIs keeping all significant correlations (significance level controlled at *α* = 0.05) and call this reference framework NO-DOWNSAMPLING.

### B. **Additional Details on the SVC Algorithm and Implemented Kernel**

In this appendix we provide additional computational details on the Support Vector Clustering algorithm we introduced in Section 2.2.1 of the paper and describe more extensively the computation of the spatiofunctional kernel.

The SVC algorithm clustering strategy consists of two steps: (a) estimation of the domain support of the sample vectors [34] to find the minimal enclosing hypersphere; (b) segmentation in disconnected regions by means of the boundaries of the domains [33].

*(a) Support Vector Domain Description*. Assume a samples set $\left\{{\vec{x}}_{i}\right\}⊑X$ composed of* N* points with *X*⊑*ℜ* ^*d*^ representing the input space and *ϕ* a nonlinear mapping function from *X* → *ℜ* ^*d*^ with associated kernel function* K* [64], such that $K\left({\vec{x}}_{i},{\vec{x}}_{j}\right)=\phi \left({\vec{x}}_{i}\right)\cdot \phi \left({\vec{x}}_{j}\right)$. The sample set domain support in the mapped space can be described by the minimal enclosing hypersphere which can be found by solving the following optimization problem:

(B.1) min R2+C∑j=1Nεjsubject  to ϕx→j−a→2≤R2+εj εj≥0; ∀j∈1,…,N

with ‖·‖ indicating Euclidean norm, $\vec{a}$ the center of the hyper sphere, and* R* its radius, and *ε*_*j*_ ≥ 0 are slack variables allowing for soft boundaries;* C* is a constant controlling the weight of outliers in the optimization problem.

The optimization problem in formula (B.1) has the following associated Lagrangian:

(B.2) L=R2−∑j=1NR2+εj−ϕx→−a→2βj−∑j=1Nεjμj+C∑j=1Nεj,

where *β*_*j*_ ≥ 0  and *μ*_*j*_ ≥ 0  are Lagrange multipliers associated with the constraints of the problem. The function* L* must be minimized with respect to the variables* R*, $\vec{a}$, and *ε*_*j*_ and maximized with respect to Lagrange multipliers *β*_*j*_ and *μ*_*j*_. The Karush-Kuhn-Tucker (KKT) [31] allows for the problem to be written in the Wolfe dual formulation of the problem

(B.3) $$\begin{array}{c} W=\overset{N}{\underset{j=1}{\sum }}\beta_{j}\phi {\left(\;{\vec{x}}_{j}\;\right)}^{2}-\overset{N}{\underset{i=1}{\sum }}\overset{N}{\underset{j=1}{\sum }}\beta_{i}\beta_{j}\phi \left(\;{\vec{x}}_{i}\;\right)\cdot \phi \left(\;{\vec{x}}_{j}\;\right) \end{array}$$

which must be maximized with respect to *β*_*j*_ subject to 0 ≤ *β*_*j*_ ≤ *C*, ∑_*j*=1_^*N*^ *β*_*j*_ = 1, for all *j* ∈ {1,…, *N*}. Data points with associated *β*_*i*_, 0 < *β*_*i*_ < *C*, lie on the hypersphere surface and are called* Free Support Vector* (FSV), while data points whose *β*_*i*_ = *C* lie outside the hypersphere surface and are referred to as* Bounded Support Vector* (BSV). Points BSV and FSV, generally called support vector, convey all information needed to define the radius* R* and the center $\vec{a}$ of the hyper sphere.

Exploiting the kernel function $K\left({\vec{x}}_{i},{\vec{x}}_{j}\right)=\phi \left({\vec{x}}_{i}\right)\cdot \phi \left({\vec{x}}_{j}\right)$ expressing dot product in the mapped space, (B.3) can be rewritten as

(B.4) $$\begin{array}{c} W=\overset{N}{\underset{j=1}{\sum }}\beta_{j}K\left(\;{\vec{x}}_{j},{\vec{x}}_{j}\;\right)-\overset{N}{\underset{i=1}{\sum }}\overset{N}{\underset{j=1}{\sum }}\beta_{i}\beta_{j}K\left(\;{\vec{x}}_{i},{\vec{x}}_{j}\;\right). \end{array}$$

For each input point $\vec{x}$, we can now reexpress its distance from the center of the hyper sphere $\vec{a}$:

(B.5) R2x→ϕx→−a→2=Kx→,x→−2∑j=1NβjKx→j,x→+∑i=1N∑j=1NβiβjKx→i,x→j.

The hypersphere radius is given by $R=\left\{R({\vec{x}}_{i})|{\vec{x}}_{i}\;\;is\;\;Free\;\;Support\;\;Vector\right\}$. The boundaries of the regions enclosing points in input space are defined by the set $\left\{\vec{x}|R(\vec{x})=R\right\}$ which can be interpreted as the set of points defining cluster boundaries.

For kernels $K(\vec{x},\vec{y})$ depending just on $\vec{x}-\vec{y}$, $K(\vec{x},\vec{x})$ is constant. For those kernel types the optimization problems reduce to

(B.6) min ∑i=1N∑j=1NβiβjKx→i,x→jsubject  to 0≤βj≤C ∑j=1Nβj=1; ∀j∈1,…,N.

*(b) Cluster Assignment*. According to [36] to assign different input vectors to different clusters a geometrical approach is exploited based on the function $R(\vec{x})$ and the observation that a generic path in input space connecting two points belonging to two different clusters is not completely enclosed in the minimal enclosing hypersphere in features space. So in such a path some points, ${\vec{y}}_{i}$, must exist such that $R({\vec{y}}_{i})>R$.

In this way, the following adjacency matrix* A* between all couples of input point ${\vec{x}}_{i}$ and ${\vec{x}}_{j}$ is defined:

(B.7) Aij=1⟺Ry→≤R∀y→∈pathx→i⟶x→j0otherwise.

Clusters are then defined as the connected components of the graph induced by matrix* A*. The above geometrical property is verified sampling 20 points on the straight path connecting each couple of points.

*Computation of the Spatiofunctional Kernel*. The spatial features used in the computation were the 3 spatial coordinates associated with each voxel after the normalization step to the MNI template in SPM. Also Talairach coordinates were associated with these voxels for identification of reference ROIs based on literature.

The resulting spatial kernel is then defined as *k*_spatial_(*v*_*i*_, *v*_*j*_) = *e* ^−∑_*k*=1,…,3_*γ*_*k*_^*S*^(*v*_*i*_(*x*_*k*_) − *v*_*j*_(*x*_*k*_))^2^^, where *v*_*i*_, *v*_*j*_ are two given voxels between which we want to calculate the kernel matrix entry, *x*_*k*=1,2,3_ ≡ (*x*_1_, *x*_2_, *x*_3_) = (*x*, *y*, *z*)_MNI_ are the voxels' coordinates in MNI space, and *γ* ^*S*_1,2,3_^ > 0 are spatial parameters controlling the clustering algorithm explained in further detail in the following section.

We now describe the functional attributes *F*_1_, *F*_2_,…, *F*_*n*_ that characterize each voxel functionally. The use of all time points' values for each voxel would lead to a high number of functional attributes (~100) with the risk of making each point a cluster in the high-dimensional mapped space. Therefore, we need to extract a low-dimensional functional representation which allows us to cluster voxels on the base of main subspace of covariance found in the data. We can obtain such a representation running a Singular Value Decomposition [36] of the time series of all voxels in output of the Multiscale Correlation Analysis. This way voxels will be clustered on the base of large scale connectivity pattern captured by main principal components. It should be noted that (i) main functional subspaces of covariance are extracted directly from data and not imposed a priori; (ii) the use of a kernel approach, which allows for nonlinear mapping of the PC weights, is supposed to grasp more complex similarities/dissimilarities in voxels functional profile and compensates for eventual inefficient PC decomposition by exploiting all information spread across components.

Given the matrix $Y=\left(\begin{array}{ccc} v_{1}^{1} & \cdots & v_{m}^{1}\\ \vdots & ∵ & \vdots \\ v_{1}^{k} & \cdots & v_{m}^{k} \end{array}\right)$ with *k* = 110 time points and* m* the number of cortical voxels in output of the correlation analysis, we calculate the decomposition

(B.8) $$\begin{array}{c} Y=ULW^{T}=\overset{r}{\underset{i=1}{\sum }}\sqrt{l_{i}}{\overset{-}{u}}_{i}{\overset{-}{w}}_{i}^{T} \end{array}$$

and associate with each voxel* v* the spatial weights ${\overset{-}{w}}_{i}^{v}$, with* i* denoting the component ordinal number. It can be shown that ${\overset{-}{w}}_{i}\propto Y^{T}{\overset{-}{u}}_{k}$ [6] so that spatial weights equal temporal scores up to a scaling factor.

To make each functional feature's contribution comparable across components we* t*-transformed the weights for each component.

Finally, the following functional kernel was defined for each couple of voxels $k_{functional}(v_{i},v_{j})=e^{-\sum_{p=1,\ldots ,P}\gamma_{p}^{F}{\left(v_{i}({{\tilde{w}}_{PC}^{}}_{p})-v_{j}({{\tilde{w}}_{PC}^{}}_{p})\right)}^{2}}$. Here again, *v*_*i*_, *v*_*j*_ are two given voxels between which we want to calculate the kernel matrix entry, $\nu_{i}\left({\tilde{w}}_{PC_{p}}\right)$ is the* t*-transformed weight for the* p*th component of the voxel* i*, and *γ*_*p*_^*F*^ are functional parameters controlling the clustering algorithm introduced in Section 2.2.2.
